# Gonadotropin Stimulation Before Sperm Retrieval in Non-Obstructive Azoospermia: Myth, Magic, or Medicine?

**DOI:** 10.1590/S1677-5538.IBJU.2026.9902

**Published:** 2026-02-27

**Authors:** Marina C. Viana, Arnold P. P. Achermann, Danilo L. Andrade, Ricardo Miyaoka, Sandro C. Esteves

**Affiliations:** 1 Clínica de Andrologia e Reprodução Humana ANDROFERT Campinas Brasil ANDROFERT – Clínica de Andrologia e Reprodução Humana, Campinas, Brasil; 2 Universidade Estadual de Campinas Faculdade de Ciências Médicas Campinas SP Brasil Programa de Pós-Graduação, Faculdade de Ciências Médicas, Universidade Estadual de Campinas (UNICAMP), Campinas, SP, Brasil; 3 Universidade Estadual de Campinas Departamento de Cirurgia, Divisão de Urologia Campinas SP Brasil Departamento de Cirurgia, Divisão de Urologia, Universidade Estadual de Campinas (UNICAMP), Campinas, SP, Brasil; 4 Aarhus University Department of Clinical Medicine Denmark Department of Clinical Medicine, Aarhus University, Denmark

## INTRODUCTION

Hormonal stimulation prior to surgical sperm retrieval in men with testicular non-obstructive azoospermia (NOA) remains one of the most debated topics in reproductive urology (1). For some, it is an unproven intervention bordering on myth; for others, its outcomes appear almost magical when sperm retrieval succeeds after years of failure. Yet, between these extremes lies medicine—rooted in physiology, supported by emerging evidence, and refined through clinical experience. This Expert Opinion synthesizes pathophysiologic principles, contemporary clinical data, and our cumulative institutional experience in managing men with NOA. We argue that hormonal stimulation—when properly indicated and individualized—represents a biologically coherent and clinically meaningful medical strategy rather than belief or chance.

## PHYSIOLOGIC BASIS: THE TESTICULAR ENDOCRINE MILIEU AND MECHANISTIC RATIONALE FOR HORMONAL MODULATION IN NOA

Spermatogenesis depends on tightly coordinated interactions between Leydig and Sertoli cells, driven respectively by luteinizing hormone (LH)-mediated testosterone production and direct follicle-stimulating hormone (FSH) stimulation (2–4). Intratesticular testosterone (ITT) concentrations are 50–100 times higher than serum levels and are indispensable for meiosis, spermiogenesis, and Sertoli-cell metabolic support (5).

In men with NOA, this endocrine equilibrium is frequently disrupted, as illustrated by the increased incidence of biochemical hypogonadism in this population (6). Although basal gonadotropin levels may be elevated, coordinated pulsatility of gonadotropin-releasing hormone (GnRH) and downstream gonadotropins may be dysregulated or insufficient to sustain optimal intratesticular endocrine dynamics (4, 7). The result is often a suboptimal microenvironment within the seminiferous tubules, leading to arrested or incomplete germ-cell maturation.

**Figure 1:**
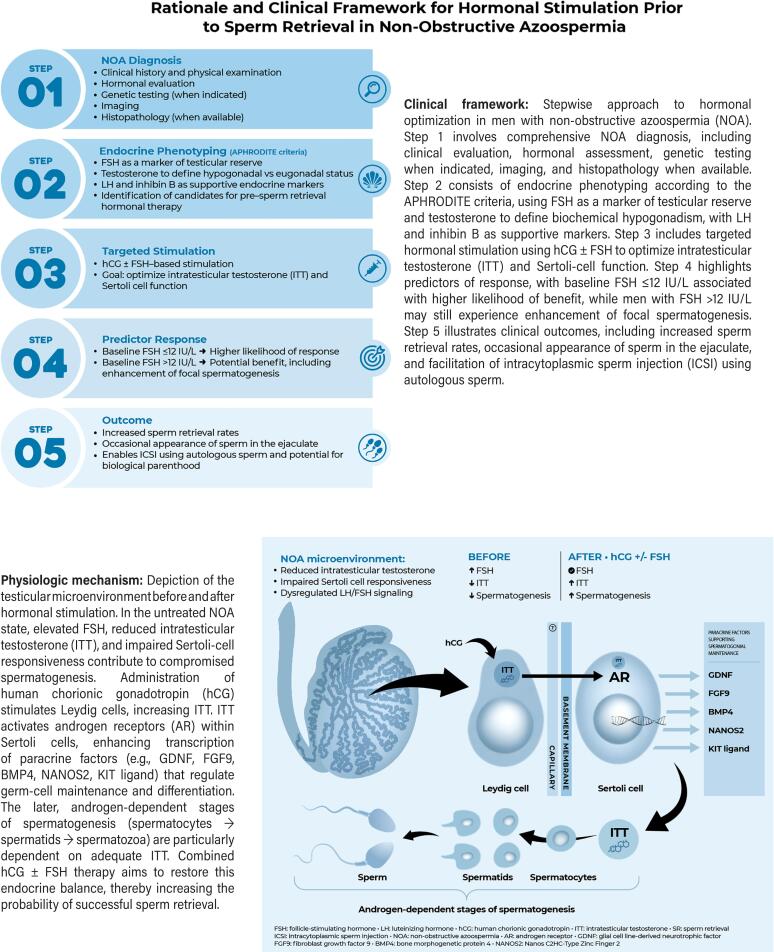
Rationale and clinical framework for hormonal stimulation prior to sperm retrieval in non-obstructive azoospermia.

The mechanistic rationale for hormonal modulation is illustrated in Figure-1. In the untreated NOA state, elevated FSH often reflects impaired Sertoli-cell reserve, while reduced ITT compromises androgen receptor (AR)-mediated transcriptional activity within Sertoli cells. Because the later stages of spermatogenesis—from spermatocytes to spermatids and mature spermatozoa—are strongly androgen-dependent, insufficient ITT may critically limit progression through these stages.

Stabilization of ITT is central to preserving the androgen-dominant intratubular environment required for germ-cell survival and differentiation. Human chorionic gonadotropin (hCG) acts as an LH analogue and directly stimulates Leydig-cell LH receptors, enhancing ITT production beyond what may be achievable endogenously in men with Leydig-cell dysfunction or impaired hypothalamic–pituitary signaling, thus restoring androgenic signaling within the seminiferous tubules (8, 9).

FSH, in turn, acts directly on Sertoli cells to support proliferative capacity, promote inhibin B secretion, regulate tight-junction dynamics, and enhance androgen-binding protein expression (8–10). Chronic exposure to elevated endogenous FSH—common in NOA patients with primary testicular failure—may induce Sertoli-cell desensitization and downregulation of FSH signaling pathways (11, 12). Experimental and clinical observations suggest that transient suppression of chronically elevated endogenous FSH, followed by controlled re-stimulation with exogenous FSH, may restore Sertoli-cell function, as reflected by increases in inhibin B and potential improvement in spermatogenic activity (13).

When hCG stimulation is combined with exogenous FSH, Sertoli-cell stimulation may be enhanced, potentially reactivating residual spermatogenic foci. Improved AR activation promotes expression of paracrine factors such as glial cell line–derived neurotrophic factor (GDNF), fibroblast growth factor 9 (FGF9), bone morphogenetic protein 4 (BMP4), NANOS2, and KIT ligand—key regulators of germ-cell maintenance and differentiation. Accordingly, tailored gonadotropin stimulation may optimize the seminiferous microenvironment and facilitate germ-cell progression through mitotic and meiotic stages. Importantly, FSH alone cannot sustain spermatogenesis without adequate ITT; however, in combination with hCG, it may synergistically enhance Sertoli-cell responsiveness.

This mechanistic insight provides biological plausibility for combined hCG–FSH stimulation even in men with elevated baseline FSH levels, provided an FSH reset is achieved after hCG administration and residual Sertoli-cell function persists (14, 15). Taken together, these observations establish a coherent physiologic rationale: hormonal therapy in NOA does not create spermatogenesis de novo but seeks to reactivate and stabilize residual spermatogenic foci by reconstructing an optimized Leydig–Sertoli endocrine milieu.

## THERAPEUTIC STRATEGIES AND DOSING CONSIDERATIONS

Given the heterogeneity of NOA and the absence of high-quality randomized trials, hormonal stimulation dosing regimens vary among centers. Most strategies are extrapolated from established protocols used in hypogonadotropic hypogonadism. Typically, hCG is administered at 1,000–3,000 IU subcutaneously or intramuscularly two to three times weekly, with dose titration guided by serum testosterone levels and clinical response. When added, exogenous FSH—either recombinant or highly purified urinary-derived FSH—is initiated at 75–150 IU two to three times weekly and adjusted according to hormonal dynamics, including inhibin B and FSH levels.

Therapy is generally maintained for three to six months, spanning at least one complete spermatogenic cycle before surgical sperm retrieval. Although largely empirical, this approach remains biologically coherent, aiming to stabilize ITT, enhance Sertoli-cell function, and improve the probability that microdissection testicular sperm extraction (micro-TESE) will identify viable spermatogenic foci.

## HOW WE DO IT: A HORMONE-GUIDED OPTIMIZATION PROTOCOL

**Figure 2:**
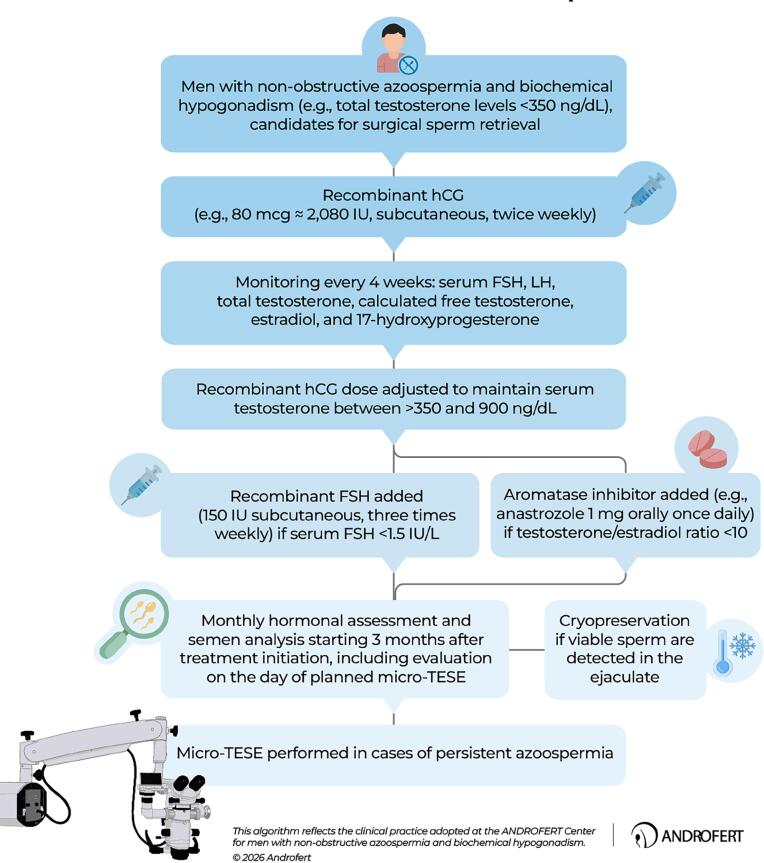
Gonadotropin-based treatment algorithm for men with non-obstructive azoospermia and biochemical hypogonadism.

At our center, we employ a structured, physiology-driven protocol to maximize sperm retrieval success in carefully selected NOA patients. Treatment begins with recombinant hCG (80 μg ≈ 2,080 IU) administered twice weekly. Hormonal profiles—including FSH, LH, testosterone, and estradiol—are assessed every four weeks. Achieving and maintaining eugonadal testosterone serum levels (350–900 ng/dL) serves as a pragmatic surrogate for adequate ITT (Figure-2).

Endocrine optimization protocol developed and utilized at the ANDROFERT Clinic prior to surgical sperm retrieval in men with non-obstructive azoospermia (NOA) and biochemical hypogonadism (total testosterone <350 ng/dL). Treatment begins with recombinant human chorionic gonadotropin (hCG), with dose titration to maintain serum testosterone within the eugonadal range (≈350–900 ng/dL). Hormonal monitoring is performed every four weeks. Recombinant FSH is added if serum FSH falls below 1.5 IU/L, and an aromatase inhibitor is introduced when the testosterone-to-estradiol ratio is <10. Semen analysis is initiated after three months of therapy; sperm detected in the ejaculate should be cryopreserved. Microdissection testicular sperm extraction (micro-TESE) is performed in cases of persistent azoospermia.

If FSH levels decline below 1.5 IU/L during hCG monotherapy—reflecting excessive suppression of endogenous gonadotropins and potential under-stimulation of Sertoli cells—recombinant FSH is introduced at 150 IU subcutaneously three times weekly. Anastrozole (1 mg daily) is added when the testosterone (ng/dL)-to-estradiol (pg/mL) ratio falls below 10 to prevent aromatase-mediated androgen depletion.

Monthly endocrine monitoring is maintained. Semen analysis begins after three months of therapy to detect possible reappearance of sperm in the ejaculate and is repeated on the day of planned sperm retrieval. In cases of persistent azoospermia despite hormonal optimization, which occurs in the vast majority of patients, micro-TESE is performed due to its superior capacity to identify isolated spermatogenic foci compared to conventional TESE. This individualized approach emphasizes endocrine reconstruction as a means of improving surgical outcomes.

## FROM PHYSIOLOGY TO RESEARCH PRIORITY

The importance of endocrine modulation in male infertility has been underscored by the Fertility and Sterility "Top Priorities for Male Infertility Research" initiative, which explicitly asked whether endocrine stimulation can improve sperm retrieval outcomes (1). This question reflects growing recognition that testicular endocrine optimization may influence not only spermatogenic health but also sperm retrieval success and downstream assisted reproductive outcomes.

The clearest precedent comes from hypogonadotropic hypogonadism (HH), in which combined hCG and FSH therapy can reliably restore spermatogenesis and lead to natural conception (16–18). Although HH represents a distinct pathophysiologic entity, it demonstrates that appropriately targeted hormonal replacement can reinitiate sperm production—even after prolonged azoospermia (19). This precedent supports a biologically plausible hypothesis: if profound gonadotropin deficiency is reversible, milder endocrine dysfunctions frequently observed in NOA may also be modifiable (18, 20). Moreover, hypogonadal NOA patients exhibit lower sperm retrieval rates than eugonadal counterparts (6, 21), suggesting that intratesticular androgen status may influence surgical success.

## CLINICAL EVIDENCE

### Meta-Analysis

In 2022, Tharakan et al. published a systematic review and meta-analysis evaluating preoperative hormonal therapy in NOA (20). Pooled data from 10 controlled studies using various types of hormonal stimulation (hCG, selective-estrogen receptor modulators, aromatase inhibitors), including 1706 individuals, demonstrated a significant and clinically meaningful increase in sperm retrieval rates among hormonally treated men (odds ratio [OR]: 1.96, 95% confidence interval [CI]: 1.08–3.56, p=0.03), particularly in those with baseline FSH ≤12 IU/L (five studies, OR 2.13, 95% CI: 1.10–4.14, p=0.02). Despite heterogeneity across regimens and study designs, the overall signal suggested that endocrine optimization may benefit selected patients with residual testicular function.

### Pilot Gonadotropin Study

In collaboration with Danish colleagues, we conducted a pilot study involving NOA patients with prior failed testicular sperm aspiration (TESA) (14). Following combined hCG ± FSH therapy, 4 of 8 men achieved viable sperm suitable for assisted conception, including two with sperm in the ejaculate and two with successful repeat TESA procedures, resulting in four live births via ICSI. Although small, this clinical signal supports the concept that endocrine modulation can convert prior failure into success in selected cases.

### Contemporary Systematic Review

Our 2023 systematic review encompassing nearly 4,000 patients demonstrated a 6% absolute increase in sperm retrieval success with hormonal therapy, corresponding to a number needed to treat of 17 (95% CI 10.5 to 39.3) (9). Additionally, approximately 6% of men developed sperm in the ejaculate. These findings highlight both promise and heterogeneity, reinforcing the need for structured phenotyping frameworks such as APHRODITE (22).

The controlled studies evaluating gonadotropin-based hormonal therapy prior to surgical sperm retrieval in NOA are summarized in Table-1. These investigations span more than two decades and include heterogeneous patient populations (normogonadotropic and hypergonadotropic phenotypes, men with prior failed TESE, and selected subgroups such as Klinefelter syndrome), diverse stimulation regimens (hCG alone, hCG combined with FSH or hMG, highly purified FSH, and mixed gonadotropin protocols), and variable treatment durations ranging from 2 to 9 months (7, 15, 23–30).

**Table 1:** Characteristics and key outcomes of controlled studies primarily evaluating gonadotropin-based hormonal therapy before sperm retrieval in non-obstructive azoospermic men.

Study / Country / Design [ref.]	Population & Key Hormonal Profile	Intervention & Duration	Control & Sperm Retrieval Method	Key Outcomes (intervention vs. control)	Adverse Events
Aydos, et al. 2003 (Turkey) RC (23)	n=108 NG	hpFSH 75 IU 3x/wk; 3 mo n=63	Yes; cTESE n=45	SR 64% vs 33%	NR
Shiraishi, et al. 2012 (Japan) RC (7)	n=48; NG+HGH; failed TESE	uhCG 5000 IU 3×/wk ± rFSH; 3–6 mo n=28	Yes; mTESE n=20	SR 21.4% vs 0% (p<0.05)	Acne 10.7%; Gynecomastia 7.1%
Hussein, et al. 2013 (Turkey) RC (24)	n=612; NG/HG; TT <300 in 140	CC-based regimens ± hCG/hMG; 3–9 mo n=496	Yes; mTESE n=116	SR ~11% vs 0%; CPR ~57% vs 33.6%	None
Cocci, et al. 2018 (Italy) PC (25)	n=50; NG	hpFSH 150 IU 3x/wk; 3 mo n=25	Yes; cTESE n=25	SR 40% vs 28% (p<0.05) CPR: 28% vs 15% (p<0.05)	NR
Hu, et al. 2018 (China) RC (26)	n=35; HG; HYPO; failed TESE	Goserelin → + hCG + hMG; 6 mo n=25	Yes; cTESE n=10	SR 8% vs 0%	40% transient sexual AEs
Amer, et al. 2019 (Egypt) RC (27)	n=1395; HG	Mixed regimens; 3–9 mo n=426	Yes; mTESE n=969	SR 27.7% vs 34.3% (NS)	NR
Amer, et al. 2020 (Egypt) PC (28)	n=40; HG; failed mTESE	Testosterone → + hCG + FSH; 4 mo n=20	Yes; mTESE n=20	SR 10% vs 0%	NR
Guo, et al. 2020 (China) RC (29)	n=184; HG; KS	uhCG 2000 IU Q2D; 3 mo n=134	Yes; mTESE n=50	SR 43.3% vs 44%; LBR similar	NR
Peng, et al. 2022 (China) RC (30)	n=569; HG	hCG 2000 IU Q2D ± uFSH; 2–3 mo n=395	Yes; mTESE n=174	SR 31.2% vs 19.5% (p=0.006)	NR
Esteves & Achermann 2024 (Brazil) RC (15)	n=616; TT <350; NG & HG	rhCG 2080 IU 2×/wk ± FSH ± AI; 3–8 mo n=291	Yes; mTESE n=325	SR 62.5% vs 51.4% (p=0.005)	Mild injection reactions (10.3%)

AI: aromatase inhibitor; CC: clomiphene citrate; CPR: clinical pregnancy rate; cTESE: conventional testicular sperm extraction; FSH: follicle-stimulating hormone; hCG: human chorionic gonadotropin; HG: hypergonadotropic; hMG: human menopausal gonadotropin; hpFSH: highly purified human-derived follicle-stimulating hormone; IU: international units; KS: Klinefelter syndrome; LBR: live birth rate; mo: month[s]; mTESE: microdissection testicular sperm extraction; NG: normogonadotropic; NR: not reported; NS: non-significant; PC: prospective; RC: retrospective; rFSH: recombinant follicle-stimulating hormone; rhCG: recombinant human chorionic gonadotropin; SR: sperm retrieval; TT: total testosterone; uhCG: urinary human chorionic gonadotropin; wk: week[s].

Despite methodological variability and the predominance of retrospective designs, several studies report higher sperm retrieval rates in hormonally treated men compared with untreated controls, particularly in selected endocrine phenotypes (7, 23-25, 28, 30). Some cohorts also demonstrate downstream reproductive benefits, including improved clinical pregnancy rates (24, 25), while others show neutral findings, especially in more severely hypergonadotropic populations or syndromic cases such as Klinefelter syndrome (27, 29).

Importantly, adverse events were generally mild and infrequent across studies. Collectively, these data suggest the existence of a biological signal supporting endocrine optimization in selected NOA patients, while simultaneously underscoring the need for prospective, phenotype-stratified randomized trials.

## APHRODITE-BASED STRATIFICATION AND CLINICAL INSIGHTS

To address the heterogeneity of endocrine phenotypes in male infertility, the APHRODITE criteria were recently introduced as a physiology-driven framework to classify men with altered testicular function based on clinical characteristics, semen analysis results, gonadotropin dynamics and testosterone status (22). This new framework can be adopted to classify NOA men into clinically meaningful distinct endocrine phenotypes.

When applied to NOA men, the framework distinguishes patients with:

Group 2: FSH and testosterone levels within normal ranges (primary spermatogenic dysfunction without biochemical hypogonadism, typically seen in cases exhibiting maturation arrest on histopathology);

Group 3: FSH levels within normal ranges associated with biochemical hypogonadism (suggesting insufficient Leydig-cell activity despite preserved Sertoli-cell signaling);

Group 4: Elevated FSH with biochemical hypogonadism or compensated testosterone levels (combined Sertoli and Leydig dysfunction, representing more advanced testicular failure).

Collectively, men with NOA may span APHRODITE groups 2, 3, and 4, reflecting the biological diversity underlying this diagnosis. This heterogeneity may partly explain why hormonal stimulation yields variable clinical results across studies.

In our cohort of more than 600 NOA patients with biochemical hypogonadism, hormonal stimulation with exogenous gonadotropins—using the protocol depicted in Figure-2—significantly improved endocrine parameters and sperm retrieval rates (15). Approximately 80% of treated individuals reached an ‘eugonadal’ state (total testosterone levels >350 ng/dL) after hormonal stimulation. Treatment also promoted an FSH reset in most individuals. Pre-micro-TESE FSH levels were significantly lower (4.0 IU/L; 95% CI: 2.9-7.6) in patients with positive micro-TESE outcomes than those with failed retrievals (8.1; CI: 5.4-15.0; p<0.0001), supporting the idea that hCG may improve Sertoli cell function by counteracting FSH receptor desensitization. Importantly, sperm retrieval rates reached 62.5% in treated men versus 51.4% in untreated controls (p=0.006), with hormonal therapy emerging as an independent predictor of micro-TESE success (adjusted OR: 2.54; 95% CI: 1.64-3.93; p<0.0001) (15). When stratified according to APHRODITE, treatment benefit was not uniform. Patients classified as Group 3 (baseline FSH levels ≤12 IU/L with biochemical hypogonadism) demonstrated superior outcomes (67.9% vs. 50.1%; adjusted OR 3.20, 95% CI 1.59-6.44, p=0.001) compared to Group 4 (elevated FSH with biochemical hypogonadism) (58.1% vs. 51.9%; adjusted OR 1.46, 95% CI 0.74-2.88, p=0.27).

This distinction is physiologically intuitive. Group 3 patients likely retain relatively preserved Sertoli-cell reserve, as reflected by normal FSH levels, while exhibiting potentially reversible Leydig-cell insufficiency. In contrast, Group 4 patients exhibit combined Sertoli and Leydig dysfunction, suggesting more advanced testicular impairment and reduced capacity for endocrine rescue. These findings reinforce a central message: hormonal stimulation should not be applied indiscriminately in NOA. Instead, endocrine phenotyping—such as that provided by the APHRODITE framework—may help identify men with residual functional reserve who are most likely to benefit from preoperative hormonal optimization.

## PHARMACOGENOMICS AND DIFFERENTIAL RESPONSE TO GONADOTROPIN THERAPY

Inter-individual variability in response to hormonal stimulation may partly reflect genetic differences in gonadotropin signaling pathways. Functional polymorphisms in the FSH β-subunit gene (FSHB), particularly the −211 G>T promoter variant, are associated with reduced transcriptional activity and lower circulating FSH levels (31). Enrichment of this variant has been reported among infertile men, including azoospermic cohorts (32). Carriers may exhibit impaired endogenous FSH signaling despite apparently normal serum levels, potentially rendering them more responsive to exogenous FSH supplementation than to tablets such as selective estrogen receptor modulators and aromatase inhibitors.

Similarly, common FSH receptor (FSHR) polymorphisms—such as Asn680Ser and Thr307Ala—have been linked to differences in receptor sensitivity and intracellular signaling efficiency (33). Although data in male infertility remain heterogeneous, these variants may influence Sertoli-cell responsiveness to both endogenous and exogenous FSH.

These genetic insights offer a biologically plausible explanation for heterogeneous treatment responses. While routine genotyping is not currently standard practice, pharmacogenetic stratification represents a logical extension of precision andrology and warrants evaluation within prospective, APHRODITE-based clinical trials, particularly in NOA males.

## FROM MYTH TO MEDICINE

When viewed collectively, physiologic rationale, meta-analytic data, and contemporary clinical evidence converge on a consistent conclusion: hormonal stimulation before sperm retrieval in NOA is neither myth nor magic—it is medicine. Its benefits are not universal, and patient selection remains essential. However, dismissing endocrine optimization outright ignores both biology and accumulating clinical data. The future lies not in debating whether hormones should be used, but in identifying which patients are most likely to benefit.

## FUTURE DIRECTIONS

Future investigations should:

- Conduct prospective multicenter randomized trials with live birth as the primary endpoint
- Integrate biomarkers of testicular reserve and endocrine responsiveness
- Incorporate pharmacogenetic stratification
- Standardize treatment response definitions
- Evaluate cost-effectiveness relative to immediate surgical intervention

## CONCLUSIONS

The journey of hormonal stimulation in NOA has evolved from skepticism to scientific plausibility. With advancing physiologic insight and growing clinical evidence, endocrine optimization should be regarded as a targeted medical strategy rather than conjecture. In selected men—particularly those with biochemical hypogonadism or low-normal FSH—reconstructing the testicular endocrine milieu may meaningfully improve sperm retrieval outcomes and expand reproductive opportunities.

## Data Availability

All data generated or analysed during this study are included in the published article.
